# Implementing Lung Ultrasound in the Outpatient Management of COVID-19 Pneumonia: A Pilot Study to Update Local Guidelines

**DOI:** 10.3389/fmed.2021.774035

**Published:** 2021-11-26

**Authors:** Chloé Chevallier Lugon, Aileen Kharat, Paola M. Soccal, Idris Guessous, Hervé Spechbach, Julien Salamun

**Affiliations:** ^1^Department of Primary Care Medicine, Geneva University Hospitals, Geneva, Switzerland; ^2^Department of Pulmonary Medicine, Geneva University Hospitals, Geneva, Switzerland

**Keywords:** COVID-19, lung, ultrasound, outpatient, pneumonia

## Abstract

**Background:** Lung ultrasound (LUS) has a good performance with a high sensitivity and specificity for the diagnosis of pneumonia compared with chest X-ray, and it has been extensively used to assess patients during the COVID-19 pandemic. This study aims to evaluate the potential advantages of the regular use of LUS for the assessment of the severity and prognosis of COVID-19 pneumonia and to propose an adapted protocol with its inclusion in current local validated and published guidelines.

**Methods:** This is a single-center prospective study conducted during the first (April–May 2020) and second (October 2020–January 2021) waves of the SARS-CoV2 pandemic in Switzerland. All adult patients presenting to dedicated test centers with a suspicion of mild-to-moderate COVID-19 pneumonia and not requiring hospitalization at the time of diagnosis were included. Patients with confirmed COVID-19 pneumonia were referred to an ambulatory follow-up unit at our institution for reassessment, with the inclusion of the use of LUS in a random selection. Descriptive statistics were calculated for demographics using percentages, means, and standard deviations according to the distribution of variables.

**Results:** Eighty-eight ambulatory patients with a confirmed COVID-19 pneumonia were included (men = 57 [59%]; mean age, 52.1 ± 13.5 years). Among these, 19 (21%) were hospitalized and none died. Twenty-five lung assessments by ultrasound were performed during the follow-up consultation. All were consistent with the clinical examination and confirmed the clinician's opinion.

**Conclusion:** The use of a standardized pleuro-pulmonary ultrasound protocol for ambulatory patients with COVID-19 could help to reduce the use of chest X-rays and improve overall management at the time of referral and eventual follow-up. However, a specific study including LUS in a systematic approach should be performed to evaluate the outcome of patients according to findings.

## Introduction

The last decade has seen the emergence and widespread use of lung ultrasound (LUS), first in the intensive care unit and then followed by the emergency setting. LUS has a good performance for the diagnosis of pneumonia with a high sensitivity and specificity compared with chest X-ray (1, 2). It has also proven to be an efficient diagnostic tool in the work-up of pneumonia diagnosis in the emergency department (3). In a metaanalysis, the pooled sensitivity and specificity for the diagnosis of pneumonia by LUS was 94 and 96%, respectively, with both a good positive and negative likelihood ratio (4). Already described in the previous H1N1 and the H7N9 influenza infection pandemics (5, 6), the use of LUS has exploded during the current COVID-19 pandemic. Of note, a recent study using chest CT as the gold standard showed that the performance of LUS appears to be higher than chest X-ray for the diagnosis of COVID-19 pneumonia (7). LUS is easily accessible, even in triage setting, and has a good diagnostic accuracy as reported by Sorlini et al. (8).

During the pandemic, the local cantonal health authorities designated Geneva University Hospitals (Geneva, Switzerland) as the referral center for COVID-19 patients. The Department of Primary Care Medicine was in charge of organizing the initial evaluation and follow-up of outpatients suffering from COVID-19. Recommendations were published and an outpatient COVID-19 unit was set up (9). Between April 2020 and January 2021, the period corresponding to the first and second waves of the pandemic, outpatients with COVID-19 pneumonia were cared for according to local validated and published guidelines, but LUS was only performed occasionally compared with chest X-ray.

In a previous study, we reported the use of an algorithm in COVID-19 pneumonia to precisely select patients who did not require hospitalization and thus were candidates to ambulatory follow-up (9). This strategy helped to preserve the local health system and avoid overwhelming the hospital (9). The aim of the present study was to assess the use and advantages of performing a LUS on a regular base among all outpatients with SARS-CoV-2-confirmed pneumonia at the time of diagnosis and without hospitalization criteria, with the intention to propose an adapted algorithm of management for suspected COVID-19 outpatients.

## Materials and Methods

This is a monocentric prospective study conducted at the Geneva University Hospitals during the first (April–May 2020) and second (October 2020–January 2021) waves of the SARS-CoV-2 pandemic. All adult patients presenting to dedicated test centers with mild-to-moderate COVID-19 pneumonia and not requiring hospitalization at the time of diagnosis were included. COVID-19 pneumonia was defined by fever >38°C, cough, and dyspnea and confirmed by a positive SARS-CoV-2 swab (naso- or oropharyngeal) performed by reverse transcriptase-PCR. At this time, LUS was not performed as standard care according to local guidelines. Patients with confirmed COVID-19 pneumonia and presenting risk factors and/or “red flags” were referred to an ambulatory follow-up unit for reassessment of pneumonia severity, depending on the evolution of symptoms. LUS was randomly performed in a nonsystematic manner in some patients as only one operator was available to perform them. The ultrasound operator had an experience of 3 years with an average of 80 ultrasounds per year and is in the process of certification by the Swiss–French section of the Swiss Society of Ultrasonography in Medicine (Groupe Romand des Echographies). The patient journey is summarized in Supplementary Appendix 1.

Ethical approval was obtained from the local ethics committee [Cantonal Commission for Ethics and Research; protocol # CCER 2020–01518)] in April 2020. Informed verbal consent for the use of data collected at the time of diagnosis was obtained from participants by telephone. A form was then sent by message (SMS/email) for them to validate this consent in writing. Verbal consent was documented in a coded database.

### Patient Cohort and Variables Collected

Data on demographic variables (age, gender), date of symptom onset, and comorbidities were collected in the emergency department before being coded into a database. During follow-up consultations, additional symptoms were collected on a dedicated computerized form nested in the patient's regular medical records to assess the overall clinical condition of each patient. These included dyspnea graded according to the New York Heart Association classification, functional impairment based on the Eastern Cooperative Oncology Group (ECOG) scale performance status, imaging studies such as chest X-ray and LUS when performed during the consultation, and available laboratory results (mainly C-reactive protein and white blood cell count). In addition, we performed a remote follow-up by phone with all patients 30 days after diagnosis to collect data on any additional consultations related to COVID-19 (or hospitalizations within 30 days).

Data were collected prospectively and recorded in a Microsoft Excel database by the same team who did the follow-up consultation. Collected data for the statistical analysis were then coded in an Excel database stored on a protected network of Geneva University Hospitals.

### LUS Characteristics in COVID-19 Pneumonia

Diverse patterns from multiple B-lines pattern (interstitial syndrome) to consolidation (alveolar consolidation) can be observed in interstitial pneumonia assessed by LUS (2). LUS is useful not only for the diagnosis of pneumonia, but also for the response to treatment, evolution, and complication monitoring (10). In COVID-19 and viral pneumonia, typical radiological patterns found are ground glass opacities and patchy infiltrates. As lesions are frequently peripheral or subpleural, it is intuitive that ultrasound can be useful in this pathology as these localizations are accessible for examination (11). The most described LUS patterns in COVID-19 pneumonia are an interstitial pattern of patchy distribution, subpleural consolidation, and pleural irregularities (12). In the study by de Alencar et al. LUS findings were considered a good predictor of death in several COVID-19 patients (13).

In an ambulatory clinic setting, patients are relatively mobile, thus allowing an easy access to posterior regions if performing LUS in a seated position. As two-thirds of COVID-19 pneumonia cases affect these regions, it is interesting to use the “12 areas” screening protocol (14), including two posterior zones per hemithorax. We used a six-zones-per-side evaluation based on higher sensitivity, but without meaningfully increasing the duration of examination. A recent review of several small studies using LUS in COVID-19 patients reported that most used the 12 areas protocol (15). Another review on the same topic reported more than 40 studies, but with a heterogenous range of scanning techniques, areas analyzed, and scoring (16). As the proposed algorithm in our study is aimed to be used in the triage area and for orientation of follow-up, we considered that a simplified modified version of the known LUS score would be more feasible. Therefore, we chose to define an area of the thorax observed as either “normal” or “abnormal.” The definition of normal was the presence of A- lines, lung sliding, and less than three B-lines. An abnormal zone was defined as the presence of three or more B-lines, consolidation, or pleural effusion. Thus, the final range of the score was 0–12. For a given area examined, the worst pattern was considered for the allocation of a normal vs. abnormal profile. Our consultation room for follow-up was equipped with a non-portable ultrasonography machine (Sonosite X-porte, Fujifilm). Details of the protocol and machine are available in Supplementary Appendix 2.

### Outcomes

As the data used for this analysis were collected for our previous study on outpatient management with COVID-19 pneumonia (9), the primary outcome was the hospitalization rate or death within 30 days after initial diagnosis of COVID-19 pneumonia. Secondary outcomes were as follows: first, to assess the feasibility of LUS in an outpatient setting and, second, to evaluate the association between LUS findings compared with clinical findings and chest X-ray.

### Statistical Analyses

We computed descriptive statistics for demographics using percentages, means, and standard deviations, according to the distribution of variables. We used STATA software, version 7.0 (StataCorp, College Station, TX).

## Results

### Participant Characteristics

Among 91 patients suffering from COVID-19 pneumonia who consulted the ambulatory follow-up unit, one did not consent to participate and two were lost to follow-up, and thus a total of 88 patients were included in the study. For the analysis, we combined data from the first and second waves of the pandemic. The mean age of patients was higher in the second wave (46.4 ±10.7 years in the first wave vs. 56.1 ± 13.8 years in the second wave; *p* = 0.006). Considering the small sample, the distribution was not normal. Demographic and clinical characteristics of the 88 patients are presented in Table 1. When merging data from both waves, most patients were men (mean age, 52.1 ± 13.5 years); 59 (60.9%) had at least one comorbidity.

**Table 1:** Characteristics of patients with COVID-19 pneumonia.

	**All patients ***N*** = 88 (100%)**	**Non-hospitalized ***N*** = 69 (78.7%)**	**Hospitalized ***N*** = 19 (21.3%)**
**Gender**			
Male	52 (59)	41 (59.4)	11 (57.9)
Female	36 (41)	28 (40.6)	8 (42.1)
**Age (years)**			
Mean (years ± SD)	52.1 (±13.5)	49.8 (±12.9)	60.4 (±12.1)
<40	18 (20.4)	17 (24.6)	1 (5.3)
40–65	58 (66)	43 (62.4)	15 (78.9)
>65	12 (13.6)	9 (13)	3 (15.8)
**Co-morbidities and risk factors**			
Asthma	13 (14.7)	12 (17.4)	1 (5.2)
Active smoker	7 (7.9)	7 (10.1)	0 (0)
Cancer	3 (3.4)	3 (4.3)	0 (0)
Diabetes	7 (7.9)	5 (7.2)	2 (10.5)
Former smoker	5 (5.6)	4 (5.8)	1 (5.2)
Hypertension	13 (14.7)	7 (10.1)	6 (31.6)
Immunocompromised	2 (2.2)	2 (2.9)	0 (0)
Obesity	4 (4.5)	3 (4.3)	1 (5.2)
Obstructive sleep apnea	5 (5.6)	4 (5.8)	1 (5.2)
**Respiratory symptoms (NYHA scale)**			
Dyspnea I	19 (21.6)	19 (27.5)	0 (0)
Dyspnea II	30 (34.1)	27 (39.2)	3 (15.8)
Dyspnea III	36 (40.9)	23 (33.3)	13 (68.4)
Dyspnea IV	3 (3.4)	0 (0)	3 (15.8)
**ECOG performance status**			
•0	13 (14.7)	12 (17.4)	1 (5.2)
•1	19 (21.7)	19 (27.5)	0 (0)
•2	30 (34)	27 (39.2)	3 (15.8)
•3	25 (28.5)	11 (15.9)	14 (73.8)
•4	1 (1.1)	0 (0)	1 (5.2)
**Radiological examination**			
Standard chest-X ray performed	75 (85.2)	56 (81.1)	19 (100)
Interstitial infiltrate found on chest-X ray	47 (53.4)	31 (44.9)	16 (84.2)
LUS performed	25 (28.4)	23 (33.3)	2 (10.5)
Interstitial lung pattern on LUS	14 (15.9)	12 (17.4)	2 (10.5)

During follow-up, all patients reported dyspnea and 55 (62.5%) presented New York Heart Association stages II (slight limitation) or III (shortness of breath during limited activity).

Regarding the ECOG scale performance status, 13 (14.7%) had no impairment (grade 0), 19 (21.7%) had some restriction in physical activity (grade 1), 30 (34%) were capable of all self-care, but unable to carry out any work activities (grade 2), 25 (28.5%) were capable of only limited self-care and were confined to bed or a chair for more than 50% of waking hours (grade 3), and one (1.1%) was completely disabled (grade 4). None were deceased (grade 5). Regarding the hospitalization rate, 19 patients (21%) eventually needed a hospitalization and none died.

During the follow-up consultation, 25 LUS were performed on 25 patients. None were done during the first consultation in the emergency department. Thirteen (52%) showed an abnormal LU/S, mainly characterized by the apparition of three or more B-lines or worsening of an interstitial syndrome in more than two explored zones. Among these 13 patients, nine had abnormalities on the chest X-ray consistent with the LUS abnormality findings, three had a chest X-ray described as normal during the initial consultation, and two never had a chest X-ray. Twelve patients had a normal LUS during follow-up. Among these, three had abnormalities on chest X-ray at the initial consultation. The difference in LUS and chest X-ray performance could be secondary either to a difference in sensitivity depending on the technique, or due to the progression/regression of disease in the elapsed time between the examinations. Additional details are shown in Table 2. Although the number of patients who had a LUS during their follow-up consultation was small and limited further analysis, no patient with a normal LUS required hospitalization (Table 3). By contrast, three patients with a normal chest X-ray required hospitalization (Table 4).

**Table 2:** Radiological findings in patients with LUS and COVID-19 pneumonia.

**Total of patients (***N*** = 25)**	**Chest X-ray normal**	**Chest X-ray abnormal**
Lung ultrasound normal (*N* = 11)	9	2
Lung ultrasound abnormal (*N* = 14)	3	9

**Table 3:** Outcome and clinical findings according to lung ultrasound results.

	**Lung ultrasound normal (***N*** = 11)**	**Lung ultrasound abnormal (***N*** = 14)**
Ambulatory (*N* = 23)	11	12
Hospitalization (*N* = 2)	0	2

**Table 4:** Outcome and clinical findings according to chest X-ray results.

	**Chest X-ray normal (***N*** = 28)**	**Chest X-ray abnormal (***N*** = 47)**
Ambulatory (*N* = 56)	25	31
Hospitalization (*N* = 19)	3	16

### Modified Algorithm

**Figure 1:**
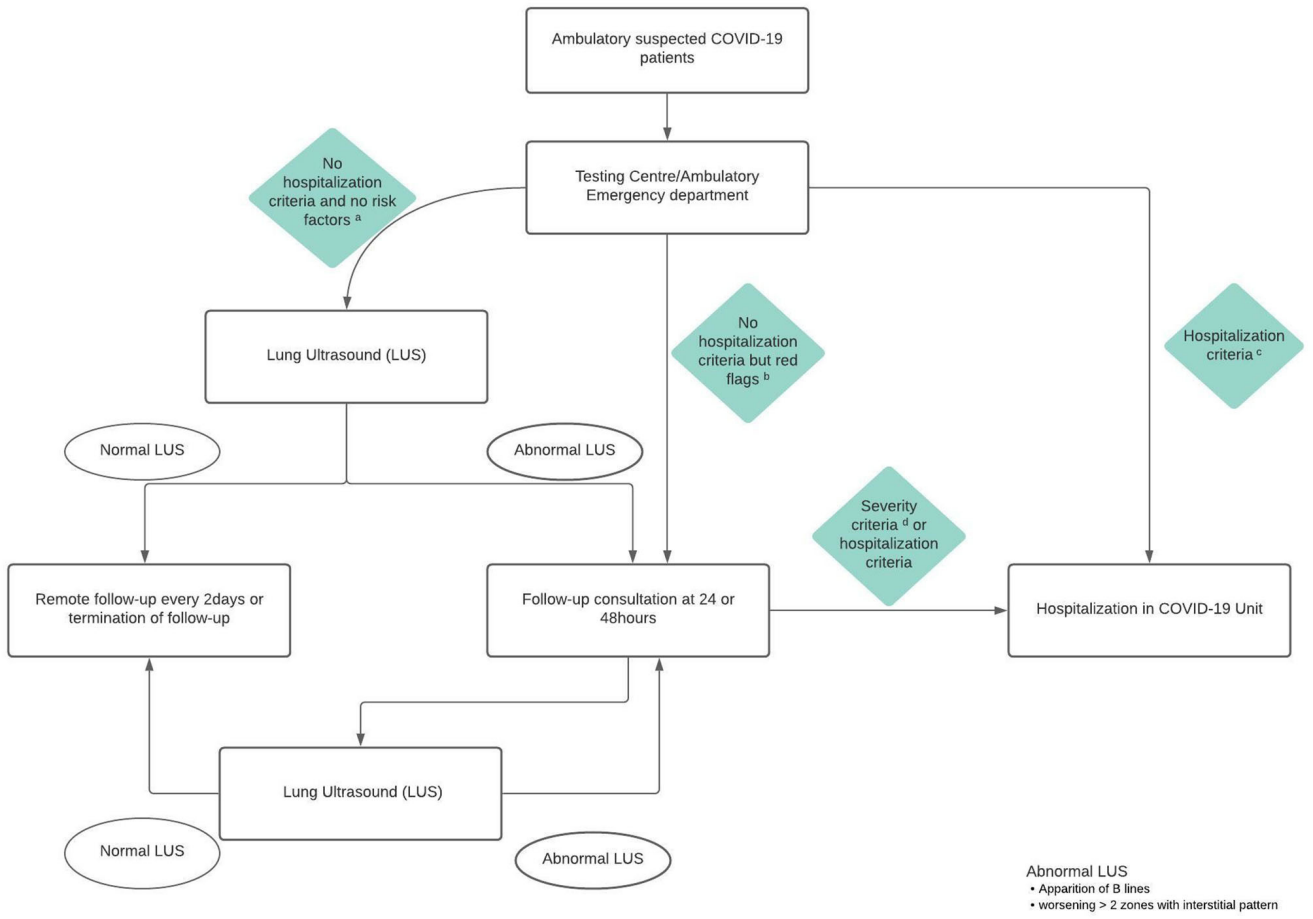
Modified algorithm for ambulatory patient management for suspected COVID-19. ^a^Risk factors: >65 years, hypertension, diabetes, cardiovascular disease, chronic respiratory disease, immunosuppression, cancer. ^b^Red flags: cough and/or fever with worsening condition, dyspnea NYHA III, hemoptysis, decreased general condition, ECOG performance status 2–3, altered state of consciousness, syncope. ^c^Hospitalization criteria: pneumonia with CURB-65 ≥ 2, oxygen dependency, sustained tachypnea (RR >20 min), decompensated comorbidity/ies). ^d^Severity criteria: audible dyspnea, inability to speak (dyspnea NYHA stage IV), serious decline in general condition (performance status >3).

Based on this experience and the available knowledge on LUS as a potential diagnostic and follow-up tool for low-risk COVID-19 patients in the ambulatory setting, we proposed a modified algorithm (Figure 1) integrating LUS as part of the standard work-up at our institution. The LUS protocol for patient evaluation at our COVID-19 ambulatory clinic is presented in Supplementary Appendix 2. LUS is therefore now associated with the clinical evaluation for the assessment of each patient.

## Discussion

The objectives of this pilot study was to investigate the potential advantages of the regular use of LUS for the assessment of the severity and prognosis of COVID-19 pneumonia and to propose an adapted protocol to be included in the institutional guidelines. Although we were unable to calculate a correlation coefficient due to the small sample size, our clinical experience showed a good association between clinical findings, LUS abnormalities, and chest X-ray results (1) as all patients with an abnormal chest X-ray had consistent LUS findings. Patients with a normal LUS and an abnormality on chest X-ray could be explained by the time gap between examinations as the latter was performed earlier, and the infiltrate visualized on the X-ray may have evolved by the time LUS was performed. During our study, patients with pathological patterns observed with LUS were more closely monitored on a 24-h or 48-h basis. Unfortunately, we were unable to perform LUS in all patients as it was not routinely available at that time. Ultrasound signs may differ, but are mainly consistent with an abnormal lung, which in the context of a pandemic and associated with respiratory symptoms are highly suggestive of COVID-19. Therefore, a normal LUS could rule out a severe COVID-19 infection and avoid unnecessary hospitalization.

The number of areas screened during LUS examination has evolved over time. After the initial proposition of three areas per hemi-thorax by Lichtenstein et al. in the BLUE protocol (17), the commonly accepted technique was an 8-area assessment recommended in the guidelines by Volpicelli et al. for interstitial syndrome (18). Nevertheless, Bouhemad et al. (19) reported a 12-point method for patients in critical care, and this method was used in the development of the LUS aeration score (20). In brief, each hemithorax was divided horizontally (superior and inferior quadrant) and three vertical zones were defined (anterior, lateral, and posterior). Fueled by the need to unify LUS practice in the current pandemic, Soldati et al. proposed a standardized LUS examination protocol for COVID-19 pneumonia (21) allowing the analysis of posterior zones, which are commonly affected in COVID-19 pneumonia. For this reason, we took the decision to perform a 12-area LUS, taking into consideration also its simplicity, mobility, applicability, and ease of teaching to physicians in charge of ambulatory patients. In addition, the integration of LUS appeared to be feasible in an ambulatory setting as the aim of our proposed adapted protocol was to systematically implement the use of LUS in the follow-up of outpatients with COVID-19 pneumonia.

Several studies have shown that LUS is a feasible and practical tool for the diagnosis of SARS-CoV-2 pneumonia (22) and could even be used as a prognostic tool (23). Although the population (nursing home residents) was different from ours (younger outpatients), this supports the use of LUS in an ambulatory setting. Guidelines have also been issued to standardize the use of LUS in pregnant women presenting with respiratory tract infections (24). A very recent multicentric study describing the use of LUS in patients with SARS-CoV-2 infection included 55 ambulatory patients and tended to show less LUS abnormalities in ambulatory patients compared with those hospitalized in an internal medicine ward or intensive care unit, with the only significant difference being the presence of large consolidations in a higher percentage of severe patients (25).

Our study has some limitations. First, the small sample size as previously mentioned. Second, we excluded potential false-negative patients in the analysis. However, patients with a negative nasopharyngeal swab, but a clinical presentation compatible with viral pneumonia, were reviewed at the follow-up visit and a second swab was taken. If the second swab was also negative, they were excluded from the analyses, thus limiting false-negatives due to repetitive testing. Third, not all patients benefited from LUS during their management and follow-up. Nevertheless, as only one experienced operator performed all the LUS examinations included in our study, we can consider that the replicability and intraobservational performance was good.

One of the major advantages of integrating LUS is to avoid the risk related to transportation of patients to the radiology department and thus the overall contamination risk. LUS is not only a potential precious diagnostic tool, but also most likely a valuable tool for the follow-up of these patients (23, 26). The availability of LUS and a trained medical team, as well as its easy accessibility, even in a triage setting, and good diagnostic accuracy (8) are all elements that favor the integration of this technique as the first-line imaging tool for the management of COVID-19 patients.

## Conclusion

Evaluation of the feasibility and added value of LUS in the ambulatory setting during the first two waves provide additional information on its potential value as a tool to determine follow-up in low-risk patients. The proposed adapted algorithm of management for suspected COVID-19 outpatients, including a LUS standardized examination protocol, may allow to replace standard radiography, and thus limit patient movement and the risk of contamination by SARS-CoV-2. This modified protocol, which is intended to be implemented in clinical practice, should be evaluated in a prospective study to confirm the clinical impression that LUS can help stratify the risk in patients with ambulatory COVID-19 pneumonia and thus allow to limit further investigations (chest X-ray, laboratory) and safely follow our ambulatory patients.

## Data Availability Statement

The raw data supporting the conclusions of this article will be made available by the authors, without undue reservation.

## Ethics Statement

The studies involving human participants were reviewed and approved by Cantonal Commission for Ethics and Research (CCER). Written informed consent for participation was not required for this study in accordance with the national legislation and the institutional requirements.

## Author Contributions

CCL, JS, and HS contributed to conception and design of the study. CCL and JS organized the database. AK designed the LUS protocol. HS performed the statistical analysis. CCL and AK wrote the first draft of the manuscript. CCL, AK, and JS wrote sections of the manuscript. IG, HS, and PS contributed to manuscript revision. All authors have read, and approved the submitted version.

## Funding

We thank the Sesam Foundation for their support. Sesam was a non-profit organization recognized as being of public utility by the Geneva cantonal authorities. It supports organizations and projects in the fields of social and humanitarian action in Geneva.

## Conflict of Interest

The authors declare that the research was conducted in the absence of any commercial or financial relationships that could be construed as a potential conflict of interest.

## Publisher's Note

All claims expressed in this article are solely those of the authors and do not necessarily represent those of their affiliated organizations, or those of the publisher, the editors and the reviewers. Any product that may be evaluated in this article, or claim that may be made by its manufacturer, is not guaranteed or endorsed by the publisher.
