# Supplementary material for: Implementing Lung Ultrasound in the Outpatient Management of COVID-19 Pneumonia: A Pilot Study to Update Local Guidelines
Source: Front Med (Lausanne). 2021 Nov 26;8:774035. doi: 10.3389/fmed.2021.774035 (PMC8660970; doi:10.3389/fmed.2021.774035)
Supplement: Supplementary file 1 [file Data_Sheet_1.PDF]

Supplementary appendix 2 (S2): Local lung ultrasound protocol for ambulatory suspected COVID-19 infection

Setting

The consultation room is composed of an examination bed, a blood pressure and pulse oximeter apparel, a computer with access to patient form and protection material composed with surgical mask, gloves and over blouse. The machine for ultrasound is inside the room with disinfection material in each room. The prevention of infection service has edited a protocol for machine and room disinfection.

The medical team responsible for patient evaluation at ambulatory setting are residents and chief resident/fellows. All operators are qualified physician having participated to a basic course on thoracic ultrasound by experienced sonographers (CCL, JS or AK). The teaching session was composed of a theoretical part and a hands-on part therefore allowing physician to acquire a systematic approach of lung ultrasound (LUS).

As described in the discussion of this article, we decided to perform a 12-zones exam based on the medical literature available and considering the good mobility of ambulatory patient (1-3). Consultation room are equipped with a machine (Sonosite X-porte Fujifilm) with a convex and linear probe. The focus will be set on the pleural line and the sub-pleural zone, because of their major interest in COVID-19 pneumonia.

Protocol

1/ The exam will be done during clinical evaluation of the patient. The estimated time for the ultrasound evaluation should be less than 10minutes to unsure fluidity of patient management in our ambulatory clinic.

2/ Patient positioning: patient will be in a sitting position on the examination table for posterior zones and lying on the back for anterior and lateral zones exploration.

3/ Image acquisition

- Convex probe in sagittal axis to have a maximal view of pleura and sub-pleural zone, on 2cm
- Description of B-lines

- B-Lines criteria :
  - Arises from pleural line
  - Vertical hyperechogenic line (laser like)
  - Extends to bottom of the screen without fading
  - Erases A lines
  - Moves with lung sliding
- Number of lines defined in a zone considered as pathological: more than 2 B-lines

- Observed zones : We chose to explore 12 zones, 6 per hemithorax (Figure 2)
  - Anterior (superior and inferior) : from sternum to anterior axillary line
  - Lateral (superior and inferior): from anterior axillary line to posterior axillary line
  - Posterior (superior and inferior): from posterior axillary line to spine
  - Superior and inferior defined as above and under the nipple

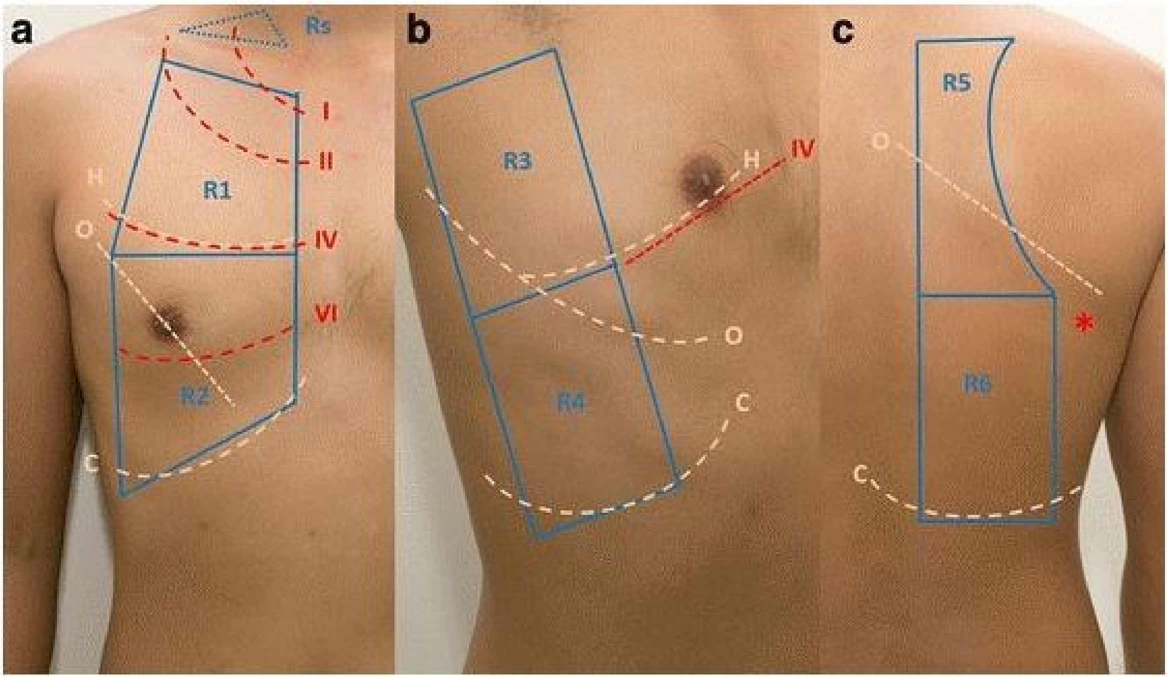

Figure 2: a. Upper and lower anterior zones, b. upper and lower lateral zones, c. upper and lower posterior zones. Images presented for right side noted R1 to R6 (4).

Every acquisition images (loop or static images) will be uploaded on the patient computerized form and a short report will be included in the medical report. This will allow comparison and follow-up. 4/Image description

To ensure inter-observer comparability and homogeneity of image interpretation, we used the definition bellow:

A. Pleural line :
   - Normal (lung sliding, regular pleural line)
   - Abnormal: irregular pleural line, pleural thickening (as described in COVID-19 Pneumonia)
B. Lung:
   - Normal (less than 3B-lines)
   - Abnormal: equal or more than 3 B-lines per field
   - Subpleural consolidation defined as interruption of the pleural line as a « dent » or « skip lesion »in the line
   - Consolidation and presence of air bronchograms
C. Pleural effusion :
   - Absent or present.
D. Pneumothorax :
   - Present or absent.

For points C and D, it is important to remind that pleural effusion is rarely found in COVID pneumonia and is suggestive of alternate diagnosis.

5/ Exam report

The report of the exam will be integrated to the patient consultation form. To optimize time, the user will just have to check pre-registered boxes in the document. If needed, complementary comments can be add.

6/ Material cleansing

As recommended by the hospital prevention infection control in our institution, probes and machine will be disinfected after each use using.

References:

1- Volpicelli G, Caramello V, Cardinale L, Mussa A, Bar F, Frascisco MF. Bedside ultrasound of the lung for the monitoring of acute decompensated heart failure. Am J Emerg Med. 2008 Jun;26(5):585-91

2- Volpicelli G, Elbarbary M, Blaivas M, Lichtenstein DA, Mathis G, Kirkpatrick AW, and al. International Liaison Committee on Lung Ultrasound (ILC-LUS) for International Consensus Conference on Lung Ultrasound (ICC-LUS). International evidence-based recommendations for point-of-care lung ultrasound. Intensive Care Med. 2012 Apr;38(4):577-91

3- Convissar DL, Gibson LE, Berra L, Bittner EA, Chang MG. Application of Lung Ultrasound During the COVID-19 Pandemic: A Narrative Review. Anesth Analg. 2020 Aug;131(2):345-350

4- Sanjan A, Krishnan SV, Abraham SV, Palatty BU. Utility of Point-of-Care Lung Ultrasound for Initial Assessment of Acute Respiratory Distress Syndrome Patients in the Emergency Department. *J Emerg Trauma Shock*. 2019;12(4):248-253. doi:10.4103/JETS.JETS_47_19
