# Supplementary material for: Implementing Lung Ultrasound in the Outpatient Management of COVID-19 Pneumonia: A Pilot Study to Update Local Guidelines
Source: Front Med (Lausanne). 2021 Nov 26;8:774035. doi: 10.3389/fmed.2021.774035 (PMC8660970; doi:10.3389/fmed.2021.774035)
Supplement: Supplementary file 2 [file Data_Sheet_2.PDF]

Supplementary appendix 1 : Patient's journey

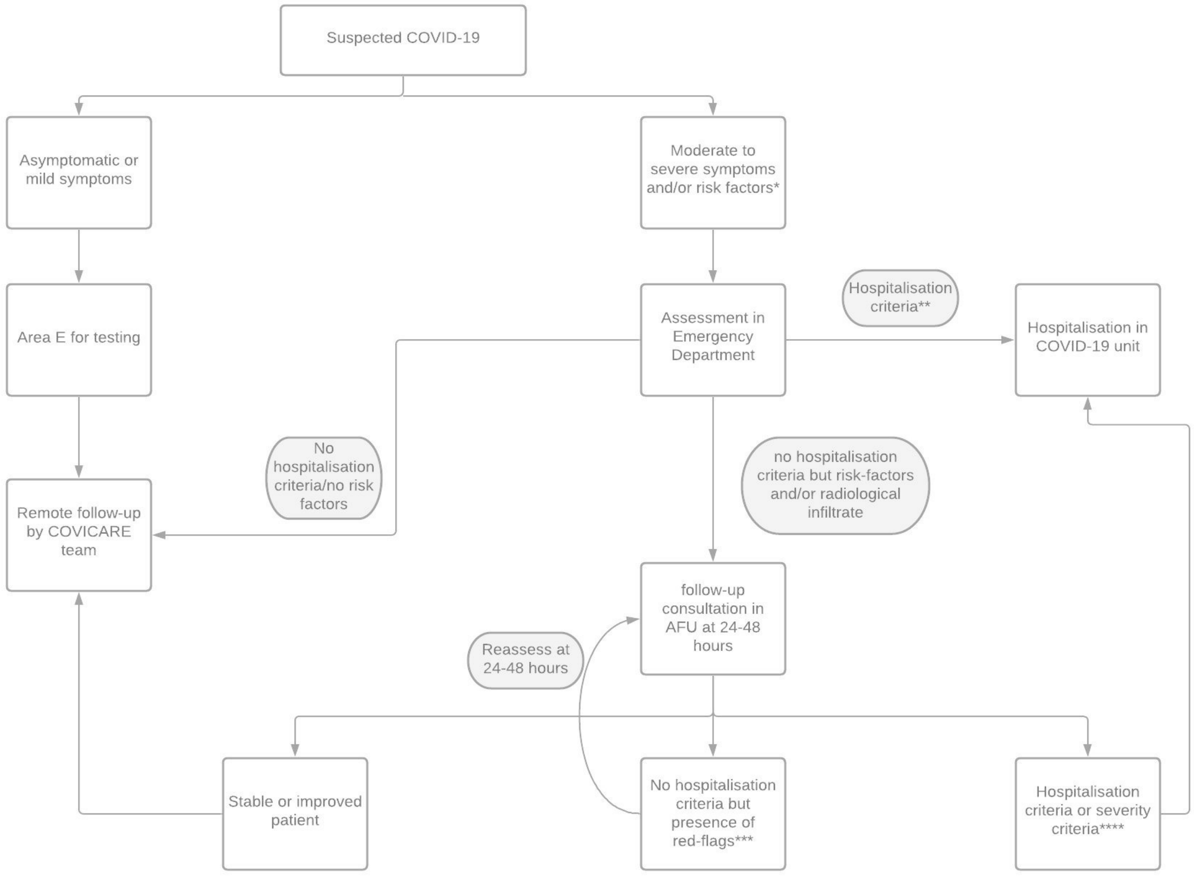

Legend: Ambulatory Follow-up Unit (AFU)

***Risk factors**: age above 65 years old, hypertension, diabetes, cardiovascular disease, chronic respiratory disease, immunosuppression, cancer.

** **Hospitalization criteria**: Pneumonia with CURB-65 > = 2, oxygen dependency, sustained tachypnea (RR≥20 min), decompensated comorbidity (ies).

*** **Red-flags**: Worsening of cough and/or fever, dyspnea NYHA III, hemoptysis, decreased general condition, ECOG performance status 2–3, altered state of consciousness, syncope.

**** **Severity criteria**: audible dyspnea, inability to speak (dyspnea NYHA Stage 4), serious decline in general condition (performance status >3).
